# A Multidisciplinary Approach to Suicide Prevention in an Outpatient Setting

**DOI:** 10.1192/j.eurpsy.2022.2175

**Published:** 2022-09-01

**Authors:** H. De Diego Ruiz, M. De Matteis, M. Mallo Caño, I. Vicente Sánchez, L. González Hernández

**Affiliations:** Hospital General Universitario Gregorio Marañón, Institute Of Psychiatry And Mental Health, Madrid, Spain

**Keywords:** suicide prevention, multidisciplinary approach, Outpatient program, community mental health center

## Abstract

**Introduction:**

The incidence of suicide is much higher in people with mental health disorders, estimating that up to 9 out of 10 people who commit suicide suffer from at least one of them. For this reason, suicide is considered by many authors as the most serious complication of psychiatric disorders. The literature and the experience of clinicians support the potential usefulness of specific measures and programs aimed at its prevention.

**Objectives:**

Congruently, throughout the last decade, consecutive strategic mental health plans in the Autonomous Community of Madrid, Spain, have included suicide prevention plans among their priorities, setting the objective of reducing suicidal behavior in the population of Madrid by implementing practical measures in the healthcare system.

**Methods:**

In the presented work we aim to summarize the multidisciplinary therapeutic process in the context of this program and the results obtained during its years of experience.

**Results:**

Retiro Community Mental Health Treatment Center launched a specific program in 2013 to meet these objectives. This initiative, that received the name PRISURE (Spanish acronym for Suicide Risk Prevention Program in Retiro), is an outpatient intervention program for immediate care, as the first appointment is scheduled within a week after referral. Intensive, comprehensive and multidisciplinary care is provided for patients with moderate to severe suicide risk.

**Conclusions:**

All professional categories that work in the Community Mental Health Treatment Center actively participate. In parallel with clinical performance, these professionals also carry out coordination tasks with other entities that are dedicated to suicide prevention, as well as with patients’ and families’ associations.

**Disclosure:**

No significant relationships.

